# First person – Lisa Elmén

**DOI:** 10.1242/dmm.046045

**Published:** 2020-07-20

## Abstract

First Person is a series of interviews with the first authors of a selection of papers published in Disease Models & Mechanisms, helping early-career researchers promote themselves alongside their papers. Lisa Elmén is first author on ‘Silencing of CCR4-NOT complex subunits affects heart structure and function’, published in DMM. Lisa conducted the research described in this article while a lab manager/research associate in Rolf Bodmer's lab at Sanford Burnham Prebys Medical Discovery Institute, La Jolla, CA, USA. She is now a scientist at Bloom Science, San Diego, CA, USA researching bacteria for the development of live biotherapeutics for neurological diseases.


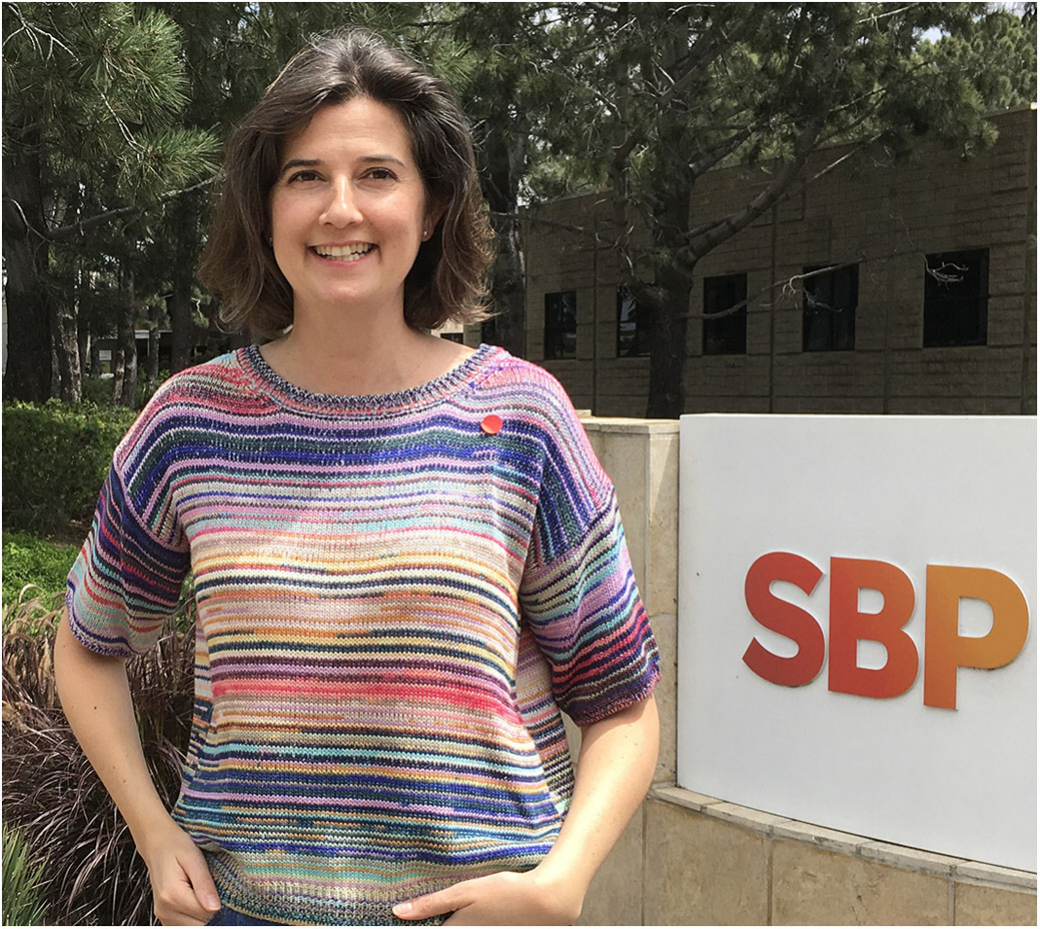


**Lisa Elmén**

**How would you explain the main findings of your paper to non-scientific family and friends?**

Genome-wide association studies (GWAS) are a big data initiative to analyze human genes, aiming to connect variations in these genes to different disease conditions. GWAS identified genetic variants close to the *CNOT1* gene that potentially could be linked to a type of heart arrhythmia that in some circumstances can be lethal. The *CNOT1* gene encodes a protein that is in the center of the CCR4-NOT complex, a group of proteins that together have an important role in regulating gene expression. Since we can't investigate this directly in humans, we used two different model systems to find out, as indicated by GWAS, if disruptions to the genes encoding this protein complex would affect the heart. We studied the effects on human heart cells in the lab, to see if silencing of these genes altered how the cells multiply and beat, and we also silenced the same genes in the heart of *Drosophila melanogaster*, the fruit fly, to see how the heart was affected in a live organism. By using these model systems, we found that reducing the gene expression of different components of CCR4-NOT indeed affects heart structure and function negatively and that silencing of some genes, including *CNOT1*, gives a stronger negative effect than others. Identifying genes that are important for heart function is needed both for preventative care and for developing new medical treatments.

**What are the potential implications of these results for your field of research?**

Our study is an example of a successful collaboration between *in silico* human genetics research and disease modeling, which hopefully will make more people take an interest in this approach. There are many reasons why this strategy is beneficial, both practical and biological. GWAS have generated many interesting findings and following up on those by combining cell-based assays that lend themselves well to high-throughput application, with a genetically well-conserved model organism like *Drosophila*, can increase the number of genetic variants investigated and reduce both time and cost as compared to rodent experiments. From a disease perspective, these model systems allow us to functionally probe the consequences of disrupting genes that in humans result in very severe disease or that may not even appear in the surveyed human population due to embryonic lethality. Based on our results, we speculate that this may be the case for CCR4-NOT; that is, only those variants that cause less severe disease appear in GWAS. This combined human genetics and disease modeling approach puts us in a better position to see the whole picture, and the more we understand the better basis we have for therapeutics development.

**What are the main advantages and drawbacks of the model system you have used as it relates to the disease you are investigating?**

We used two different model systems in this study, one *in vitro*, with cell lines and human induced pluripotent stem cells differentiated into cardiomyocytes, and one *in vivo*, using *Drosophila*. Both of these systems have distinct advantages and drawbacks, and, by combining the two, we could address different aspects of the same question. For example, the *in vitro* system allowed us to assess the effects on human cardiomyocyte proliferation, something that could not be done *in vivo* but suffers the drawback of an artificial environment. For *in vivo* experiments, we used *Drosophila*, which benefits from well-conserved genes, and while comparisons with the human heart are limited by the fly heart's different morphology and lack of a closed circulatory system, these features are what allow us to functionally assess genes of interest that the vertebrate heart does not tolerate manipulation of very well.

**What has surprised you the most while conducting your research?**

Not specifically for this paper but overall, I've been surprised over the versatility of *Drosophila* as a model organism and I'm impressed by how much we have learned about human genetics from working with this little insect.

**Describe what you think is the most significant challenge impacting your research at this time and how will this be addressed over the next 10 years?**

Basic research is very often struggling to obtain funding, and the usefulness of *Drosophila* is met with quite a bit of skepticism. This organism has been used for genetics research for more than a hundred years, and despite all it has taught us, it does not always get the credit it deserves. We have completely different resources today as compared to the last century, but we have shown in this paper that *Drosophila* is still very valuable when you combine it with more recent technology. To overall improve funding for basic research it's crucial that a general audience understands the role of basic science in paving the way for the development of therapeutics. We have a wealth of means of communication today, and I think the scientific community overall starts to see that to gain more support, we need to reach out to share our discoveries and explain how they contribute to improving human health.

**Figure:**
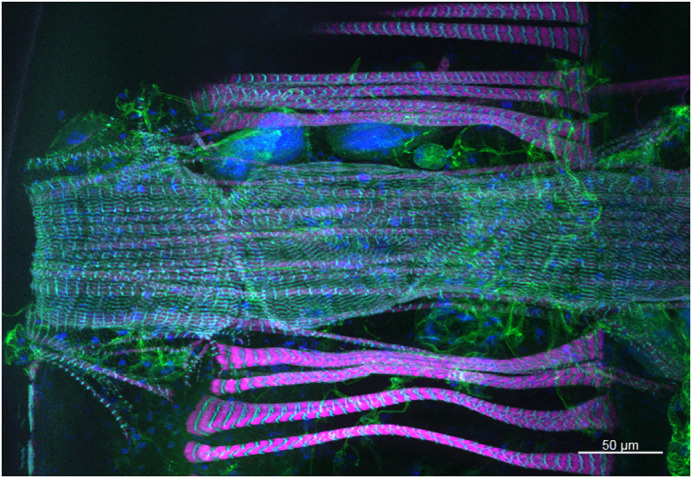
***Drosophila* heart (25×, by Katja Birker).**

**What changes do you think could improve the professional lives of early-career scientists?**

PhD students as well as postdocs could certainly benefit from help with career planning, regardless of whether the direction is toward an academic faculty position or a job in industry. A postdoc position has long been the default continuation of a PhD degree in science, and while a postdoc is a valuable next step and a necessary one if continuing in academic research, it is actually not needed for many jobs out there. To decide on what is right for you and to plan accordingly, learning early on about non-academic job opportunities and what they require, will allow you to make educated choices about your training. Likewise, if you aim to start your own lab, you need to know how to increase your chances of getting a faculty position, which may be by obtaining an early career grant for which you are only eligible within a specific time frame. I was fortunate in that Sanford Burnham Prebys Medical Discovery Institute has a department dedicated to education and training, which, through workshops and other career planning events, greatly helped me make decisions on my next steps. I really wish that this type of resource would be available to all early-career scientists, because there are so many exciting job opportunities to look into.

**What's next for you?**

Our work on the CCR4-NOT complex unfolded over many years, and in parallel with this study, I obtained a PhD degree at the same institute but in a different field. My graduate work focused on the influence of gut microbiota on cancer development, and I recently joined a startup company, Bloom Science, where I will continue to do research on bacteria with the objective of developing live biotherapeutics for neurological diseases.

“[…] the scientific community overall starts to see that to gain more support, we need to reach out to share our discoveries and explain how they contribute to improving human health.”

**How can we inspire the general public's interest and understanding of science?**

Community outreach is a great way of engaging a larger audience, whether that is by volunteering for school and community events, or by giving more formal general audience presentations. These are good opportunities to discuss your work, because the specific challenge with scientific communication is that research is a process without a clear end. Biological and medical research need many years and the collective input of independent labs to unequivocally conclude the validity of a new discovery. This makes the news and social media dissemination of new scientific findings complicated, and I would like to see scientists more in charge of the information that goes out to prevent the results from being distorted. I'd like to encourage all early-career researchers to give some thought to how you can translate your work into information that is understandable for everyone, and to maybe also get some training in scientific communication. It is also a lot of fun interacting with a general audience, and you'll get questions that you'd never get from a colleague, which can prompt some thinking outside the box.
